# Exploring the Interdisciplinary Nature of Precision Medicine：Network Analysis and Visualization

**DOI:** 10.2196/23562

**Published:** 2021-01-11

**Authors:** Xin Xu, Jiming Hu, Xiaoguang Lyu, He Huang, Xingyu Cheng

**Affiliations:** 1 General Medicine Ward Renmin Hospital of Wuhan University Wuhan China; 2 School of Information Management Wuhan University Wuhan China; 3 Department of Gastroenterology Renmin Hospital of Wuhan University Wuhan China; 4 Department of Cardiology Renmin Hospital of Wuhan University Wuhan China; 5 Department of Radiology Ezhou Central Hospital Ezhou China

**Keywords:** precision medicine, interdisciplinary, social network analysis, co-occurrence analysis

## Abstract

**Background:**

Interdisciplinary research is an important feature of precision medicine. However, the accurate cross-disciplinary status of precision medicine is still unclear.

**Objective:**

The aim of this study is to present the nature of interdisciplinary collaboration in precision medicine based on co-occurrences and social network analysis.

**Methods:**

A total of 7544 studies about precision medicine, published between 2010 and 2019, were collected from the Web of Science database. We analyzed interdisciplinarity with descriptive statistics, co-occurrence analysis, and social network analysis. An evolutionary graph and strategic diagram were created to clarify the development of streams and trends in disciplinary communities.

**Results:**

The results indicate that 105 disciplines are involved in precision medicine research and cover a wide range. However, the disciplinary distribution is unbalanced. Current cross-disciplinary collaboration in precision medicine mainly focuses on clinical application and technology-associated disciplines. The characteristics of the disciplinary collaboration network are as follows: (1) disciplinary cooperation in precision medicine is not mature or centralized; (2) the leading disciplines are absent; (3) the pattern of disciplinary cooperation is mostly indirect rather than direct. There are 7 interdisciplinary communities in the precision medicine collaboration network; however, their positions in the network differ. Community 4, with disciplines such as genetics and heredity in the core position, is the most central and cooperative discipline in the interdisciplinary network. This indicates that Community 4 represents a relatively mature direction in interdisciplinary cooperation in precision medicine. Finally, according to the evolution graph, we clearly present the development streams of disciplinary collaborations in precision medicine. We describe the scale and the time frame for development trends and distributions in detail. Importantly, we use evolution graphs to accurately estimate the developmental trend of precision medicine, such as biological big data processing, molecular imaging, and widespread clinical applications.

**Conclusions:**

This study can help researchers, clinicians, and policymakers comprehensively understand the overall network of interdisciplinary cooperation in precision medicine. More importantly, we quantitatively and precisely present the history of interdisciplinary cooperation and accurately predict the developing trends of interdisciplinary cooperation in precision medicine.

## Introduction

### Background

Precision medicine is a new medical model that tailors disease prevention and treatment by considering differences in people's genes, environments, and lifestyles [1]. The emerging field of precision medicine provides more precise, evidence-based medical services [2]. Precision medicine is currently widely used in clinical medicine, preventive medicine, and other fields [3-5]. Precision medicine also faces many challenges, such as disease heterogeneity, diverse populations, and ethical considerations [6-9].

Precision medicine has the following interdisciplinary characteristics: (1) the core technologies in precision medicine are provided by multiple disciplines, such as genomics technology, big data, and nanobiotechnology [10-13]; (2) precision medicine is widely applied in medical fields, such as internal medicine, surgery, and oncology [14-16]; (3) many difficulties and challenges still exist in the development of precision medicine that require extensive interdisciplinary cooperation [17]; (4) as is known to us, the subject categories of studies are assigned by the Web of Science to represent the disciplines involved in the research [18]. However, we discovered that the subject categories of the studies concerning precision medicine retrieved from the Web of Science are numerous, indicating that precision medicine is an exact interdiscipline.

Interdisciplinary collaboration refers to two or more involved disciplines integrating their knowledge and methods to form a new research field [19]. Historically, the emergence of interdisciplinary collaboration often indicates the development level and breakthroughs of the research field [20]. Therefore, the interdisciplinary cooperation level can represent the developmental level and trend to some extent. In addition, an investigation revealed that researchers within the fields of clinical and translational science, which is an interdisciplinary and collaborative research, need tools to process resource discovery and collaboration [21]. To date, scholars in various fields, such as information behavior research and library sciences, have explored the nature of interdisciplinary collaboration using methods such as bibliometrics and co-word analysis [22,23]. These studies help researchers and practitioners in these fields better understand the nature of interdisciplinary collaboration.

Thus far, no study evaluated interdisciplinary collaboration in precision medicine. Our study aims to use a social network analysis, a co-occurrence analysis, and visualization to objectively and quantitatively reveal the status of interdisciplinary collaboration in precision medicine and vividly exhibit the structure, pattern, duration, and evolution trend of interdisciplinary collaboration in precision medicine. This study could help scientists, clinicians, policymakers, and fund providers better understand the interdisciplinary status of precision medicine, assess its maturity, and predict future trends.

### Literature Review

Precision medicine was born during the post-Genome Wide Association Study program [24-26]. It originally targeted different populations stratified by genetic biomarkers [27]. This led to the development of precision medicine in the following broad directions. First, biomarkers are key elements of the precision medicine knowledge system and bottlenecks for clinical applications. To discover reliable biomarkers, scientists in different fields (eg, clinical medicine, genetics, chemistry, physics, pathology, and radiology) have worked closely together and have made exciting progress [28]. As a result of interdisciplinary collaboration, different types of biomarkers have been found that play important roles in diagnosis, treatment, and prognosis [29]. The second most common interdisciplinary activity is the expanding application of precision medicine. With growing awareness of the advantages of precision medicine, such as improving efficacy and reducing side effects, research on and applications of precision medicine have spread from clinical oncology to other clinical fields, such as chronic obstructive pulmonary disease, cardiovascular disease, and diabetes prevention [30,31]. However, as precision medicine is increasingly used in clinical practice, new problems related to economics, ethics, and public health must be addressed [32-34]. The collaboration of clinicians, economists, ethicists, and public health managers is an obvious feature of precision medicine research and a symbol of its maturity. Therefore, the study of the interdisciplinary nature of precision medicine will help us comprehensively understand the major applications and level of maturity of precision medicine.

Interdisciplinarity refers to traditional disciplines breaking through the boundaries of their respective knowledge systems [35]. Scientists collaborate together, and a new discipline is born. The level of interdisciplinary integration can indirectly reflect the maturity and future trends of a specific field [36].

Social network analysis is a tool used to initially investigate social structure (eg, social media networks [37], collaboration [38], and disease transmission [39]) in the field of sociology. Currently, however, scientists use social network analysis widely to evaluate collaborative interdisciplinary networks [40-42]. Social network analysis uses indexes such as points, lines, and links to accurately measure the degree of collaboration between disciplines and to comprehensively display a visualized network map, which can help researchers better understand the overall status of the interdisciplinarity of a specific field [43].

### Study Rationale

Interdisciplinarity is an important feature of precision medicine. For precision medicine researchers, health managers, and research funders, informatic research about interdisciplinary collaboration is of great significance to understanding a field’s developmental level and predicting developing trends. Thus far, however, there has been no informatic research to reveal the interdisciplinary puzzles of precision medicine. Our study uses social network analysis to explore and visualize the precise status of the interdisciplinarity of precision medicine. The significance and innovation of our research mainly include the following aspects: (1) The framework and distribution of the overall collaboration of precision medicine. (2) Which major communities exist in collaborative networks, indicating the main areas and directions of precision medicine. (3) The evolutionary trend of interdisciplinary collaboration.

## Methods

### Data Collection and Processing

**Figure 1:**
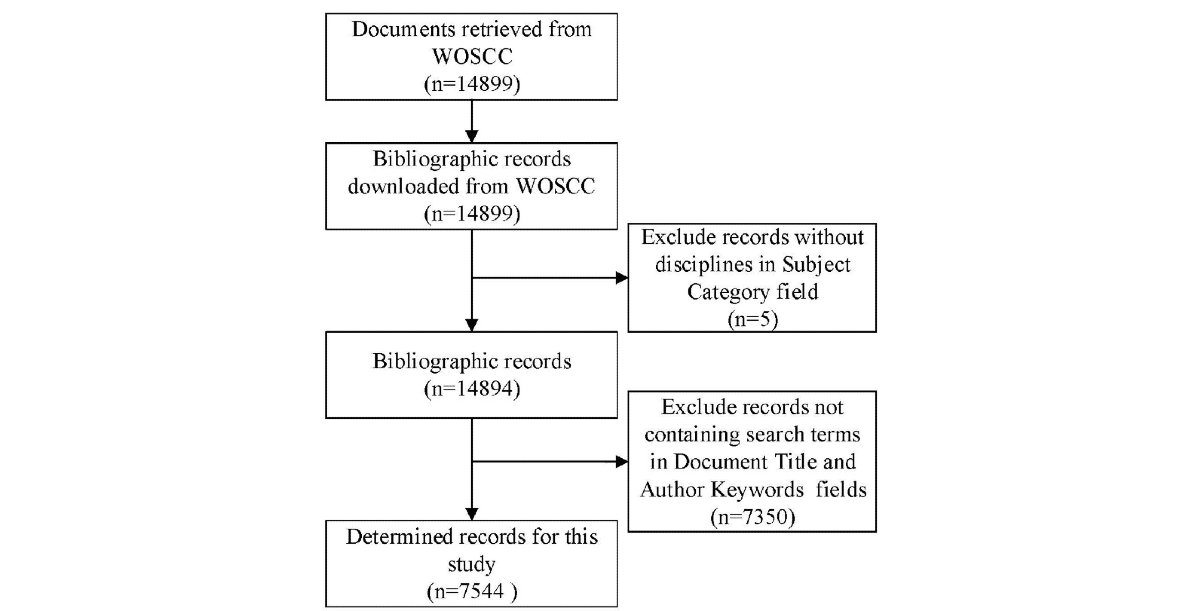
Precision medicine research search procedure for documents in the Web of Science Core Collection database. WOSCC: Web of Science Core Collection.

We used the Web of Science Core Collection, a major database that covers most major medical studies. In addition, research on precision medicine included in the Web of Science Core Collection can be considered to represent the progress of the current level of research. For maximum comprehensiveness, we searched the relevant literature in the Web of Science Core Collection with defined strategies such as searching the keywords “precision medicine,” “P4 medicine,” “personalized medicine,” and “stratified medicine” over a time span covering 2010-2019. Finally, the bibliographic data (articles, reviews, and proceedings papers) were downloaded for subsequent analysis. The data processing is as shown in Figure 1.

According to the methods of previous studies, we considered literature containing the aforementioned search terms in the title and keywords to be most relevant, while literature containing the search terms only in the abstract were less relevant to the research topic [44]. Therefore, we removed literature that contained the search terms only in the abstract and retained documents containing the search terms in the title or keywords. Moreover, we excluded documents without the subject category. The rest of the bibliographic data were qualified for the research.

The Web of Science Core Collection marks the subject category of each document in its bibliographic data. If the document is interdisciplinary, the subject category field often contains multiple subject categories. This means that the co-occurrence of subject categories in the subject category field indicates the interdisciplinary nature of a document [45,46] and reflects interdisciplinary cooperation on the issue. Therefore, we performed an in-depth analysis of the subject categories included in the subject category field to clarify the characteristics of interdisciplinary cooperation on precision medicine research.

### Methodology and Tools

#### Background

Co-occurrence theory holds that if two items appear together in the same intentional unit (such as author, keyword, institution, English), this indicates a strong correlation between the two projects, such as similar semantic connotations, interaction between the items, or cooperation [47,48]. Similarly, if multiple disciplines appear together in the subject category field of bibliographic data, we can speculate that there is cross-cooperation between these disciplines [45,49]. By extracting the cross-cooperative relationships of all subject categories, a complete cooperative network is formed. The following analysis of the structure can reveal hidden cooperation features and laws [40,50].

#### Network Analysis

An important part of co-occurrence analysis is to analyze the network structure formed by the co-occurrence relationship for the overall and individual network indicators. We introduced the bibliographic data into the Science of Science Tool，version 1.2 beta (Cyberinfrastructure for Network Science Center, Indiana University, Bloomington, Indiana, United States), to extract the subject category field, count the number of subjects, and calculate the co-occurrence frequency between any two subjects [51]. This means that two subjects appear together in the same bibliographic data. For co-occurrence, the total frequency of co-occurrence is equal to the amount of bibliographic data containing the two subjects. On the basis of extracting the discipline and its co-occurrence relationship, a cross-disciplinary cooperation network was generated and exported as a “.net” file. In the co-occurrence network file, the points and edges represent the disciplines and their cooperative relationship, respectively, and the frequency of appearance and the frequency of co-occurrence are weighted.

In general, the network analysis focuses on its largest connected subgraph because the isolated or unconnected points do not reflect the main connotation. We used SCI2 to eliminate the isolated points (disciplines) in the disciplinary cooperation network to generate the largest connected subgraph. The new net file was used as the basis for subsequent analysis. The maximum connected subpicture file was imported into Pajek [52] for network index calculation (including centrality, density, and aggregation coefficient), and a topology map of the cooperative network was generated. Network indicators (including integrated network indicators and individual network indicators) are the embodiment of the cooperative network structure, reflecting the position and function of the discipline in the cooperative network as well as the laws and trends of cross-disciplinary cooperation. We can even speculate on the laws and trends of the cooperative discipline network.

It is worth noting that the nodes in the co-occurrence network exhibit certain aggregation characteristics due to the different connection distributions. The nodes that are grouped into the same class form a community, indicating that the nodes are similar in a certain aspect. In the same way, if the disciplines in the interdisciplinary cooperation network are divided into the same class due to the cooperative relationship, this indicates that the intensity of crossing cooperation between them is strong. We can also infer that the disciplines mentioned above have unity in their research direction and theme. In this study, we used the Louvain community partitioning algorithm in Pajek [53] to divide the disciplinary cooperation network into numerous different communities and explore the characteristics of precision medicine research in terms of disciplinary cooperation.

#### Measures of Interdisciplinary Degree

Subject category and its co-occurrence relationship in Web of Science bibliographic data provide strong support to describe the extent of interdisciplinary cooperation or interdisciplinarity [45,49]. We also used String’s diversity index and the specialization index to calculate the interdisciplinary degree of precision medicine research [46,54].

String’s diversity index calculates the diversity of discipline cooperation. The greater the value is, the greater the interdisciplinary degree is. The calculation formula is as follows:


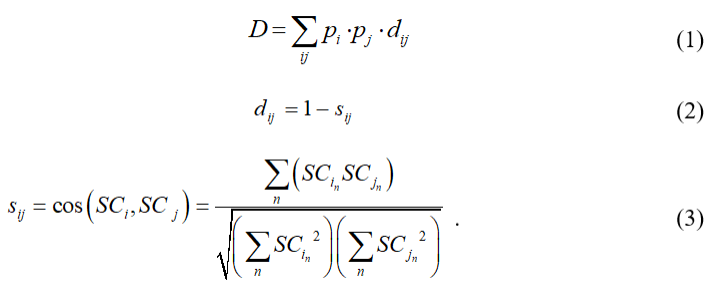



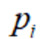
 and 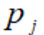 is the proportion of the occurrence frequency of subject 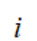 and 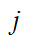 to the sum, and 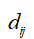
is the degree of difference between subject 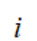 and 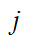; its value is calculated by Formula 2. Furthermore, 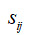 is Salton's cosine similarity between the two disciplines [55]; its value is calculated by Formula 3. Formula 3 calculates the similarity of subjects based on the number of co-occurrences between one subject and the other disciplines as well as the similarity between two associated disciplines. It can transform the disciplinary cooperation network into a co-occurrence matrix and into a cosine similarity matrix, indicating the similarity of any two disciplines.

The specialization index is used to describe the concentration level of disciplinary cooperation; its meaning is opposite to String’s diversity index. The larger the specialization index is, the fewer disciplines involved in the cooperation. This indicates that overall cooperation is limited. The formula of the specialization index is as follows:


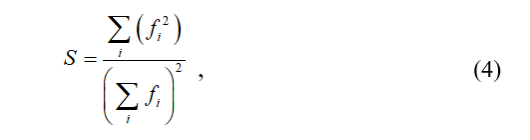


where 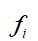 is the frequency of occurrence of subject 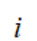.

Combining the above two indicators, the discipline cooperation status of precision medicine research can quantitatively reveal the degree of yearly cooperation of one discipline. Then, we can discover the chronological change of the disciplinary cooperation of precision medicine research.

#### Visualization and Evolution Patterns

Visualization has the advantage of displaying the co-occurrence network structure and posture, thus helping us better understand the meaning of the research object. Due to the superiority of VOSviewer [56] in terms of visualization effects, we selected it to show the subject cooperation network, including the overall network at the community level and the network of each single community. In addition, we revealed the chronological changes of subject cooperation. In this study, we divided the bibliographic data according to age and introduced Cortext to generate interconnected strip diagrams to show the chronological characteristics of interdisciplinary cooperation. Because of the various cooperation intensities and distribution structures, there were significant differences in the subject cooperation community. The diversity between the communities is reflected in the two indicators, such as density and average centrality. It can quantitatively display the relative position and development status of the cooperative community in the whole subject cooperation network. Based on the above two indicators and the sum of disciplinary frequency in the community, we drew a strategic diagram to intuitively show the relative development trend of the discipline cooperation community [57]. The strategy map uses the average of all community densities and centralities as the origin, with the centrality as the x-axis and the density as the y-axis, dividing the map into 4 quadrants to show the differences between the communities. The centrality reflects the degree of association between a community and other communities. The higher the value, the more central the community is in the entire network. The density reflects the closeness between the communities. The higher the value, the closer the internal association is and the more mature the research field is. Each community is distributed in 4 quadrants due to its centrality and density. The community in the first quadrant, with a high degree of centrality and density, is the core of the whole research and the most mature development; the community in the second quadrant, with a lower center and higher density, is not the core but is mature in the whole research. The community in the third quadrant has low centrality and density; it is neither the core of the whole research nor immaturely developed. The community in the fourth quadrant has higher centrality but lower density; it is the core of the whole study, but the development of the community is not mature. The different distribution of discipline cooperation communities in the quadrant represents their relative development status.

## Results

### Disciplines Involved in Precision Medicine Research

**Figure 2:**
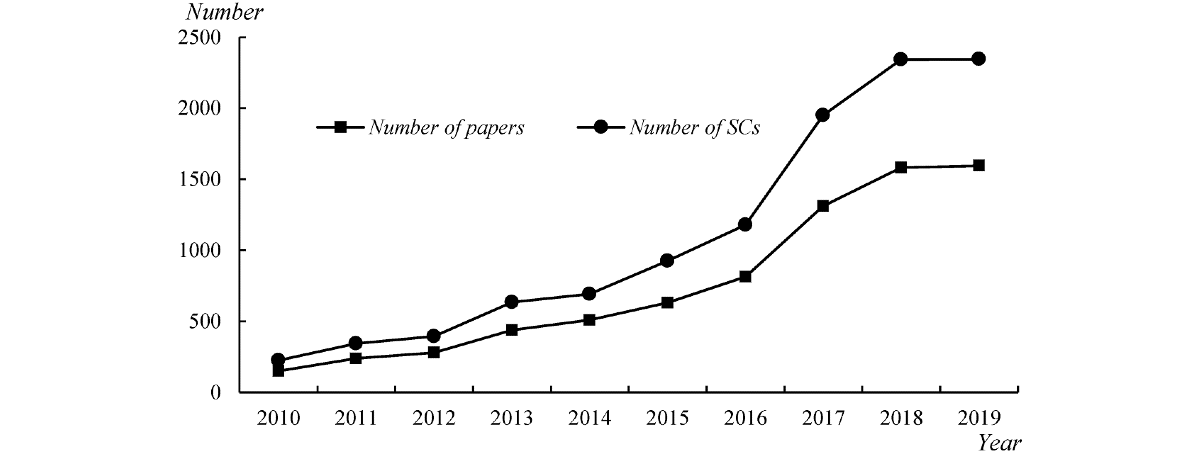
The basic statistics of precision–medicine related sample papers and subject categories from 2010 to 2019. SCs: subject categories.

In this study, we obtained a total of 7544 papers. As shown in Figure 2, while the number of precision medicine–related research papers is increasing, the number of disciplines involved is also increasing, which indicates that disciplinary cooperation in precision medicine research is constantly intensifying.

We obtained the bibliographic records of the 7544 studies from the Web of Science Core Collection, and then performed the unique statistical analyses on the subject categories field, confirming a total of 105 disciplines involved. It is surprising that the categories of disciplines mentioned above covers almost all the disciplines included in the Web of Science Core Collection.

Taking into account the actual situation of precision medicine research, we selected 75 disciplines with frequencies greater than or equal to 10 in the following analysis. By analyzing the cross-cooperation of 75 disciplines, we were able to reveal the interdisciplinary features of precision medicine research.

Table 1 lists the 75 disciplines with a frequency greater than or equal to 10. The sum of their frequency’s accounts for 98.8% of the total frequency (10,910/11,039), which largely covers all the disciplines involved in precision medicine research. However, precision medicine research focuses on disciplines such as oncology, pharmacology and pharmacy, genetics and heredity, research and experimental medicine, biochemistry and molecular biology, general and internal medicine, neurosciences and neurology, health care sciences and services, cardiovascular system and cardiology, and cell biology. Their proportion is as high as 52.8% (5833/11,039), while the remaining disciplines share the remainder of the sum, highlighting the disciplinary concentration of precision medicine research.

**Table 1:** Seventy-five disciplines with frequencies equal to or greater than 10 involved in precision medicine research.

Item	Subject category	Frequency		Item	Subject category	Frequency
1	Oncology	1457		39	Biomedical social sciences	59
2	Pharmacology and pharmacy	1199		40	Materials science	53
3	Genetics and heredity	566		41	Social sciences - other topics	50
4	Research and experimental medicine	513		42	Physiology	49
5	Biochemistry and molecular biology	485		43	Medical ethics	37
6	General and internal medicine	416		44	Nursing	36
7	Neurosciences and neurology	390		45	Microbiology	35
8	Health care sciences and services	300		46	Dermatology	34
9	Cardiovascular system and cardiology	254		47	Transplantation	33
10	Cell Biology	253		48	Biophysics	32
11	Pathology	237		49	Integrative and complementary medicine	31
12	Biotechnology and applied microbiology	232		50	Information science and library science	30
13	Mathematical and computational biology	230		51	Geriatrics and gerontology	28
14	Respiratory system	228		52	Physics	27
15	Computer science	205		53	Ophthalmology	27
16	Chemistry	204		54	Nutrition and dietetics	26
17	Medical informatics	203		55	Government and law	26
18	Psychiatry	196		56	History and philosophy of science	24
19	Engineering	193		57	Otorhinolaryngology	23
20	Science and technology - other topics	192		58	Dentistry, oral surgery, and oral medicine	23
21	Public, environmental, and occupational Health	177		59	Infectious diseases	22
22	Endocrinology and metabolism	158		60	Optics	22
23	Radiology, nuclear medicine, and medical Imaging	155		61	Reproductive biology	21
24	Mathematics	152		62	Substance abuse	16
25	Immunology	148		63	Education and educational research	14
26	Hematology	139		64	Social issues	14
27	Gastroenterology and hepatology	138		65	Telecommunications	13
28	Urology and nephrology	115		66	Instruments and instrumentation	13
29	Pediatrics	107		67	Sport sciences	12
30	Surgery	107		68	Veterinary sciences	12
31	Medical laboratory technology	97		69	Environmental sciences and ecology	12
32	Obstetrics and gynecology	96		70	Food science and technology	12
33	Toxicology	85		71	Orthopedics	12
34	Business and economics	83		72	Anesthesiology	10
35	Allergy	76		73	Anatomy and morphology	10
36	Life sciences and biomedicine - other topics	74		74	Legal medicine	10
37	Psychology	68		75	Electrochemistry	10
38	Rheumatology	64				

**Figure 3:**
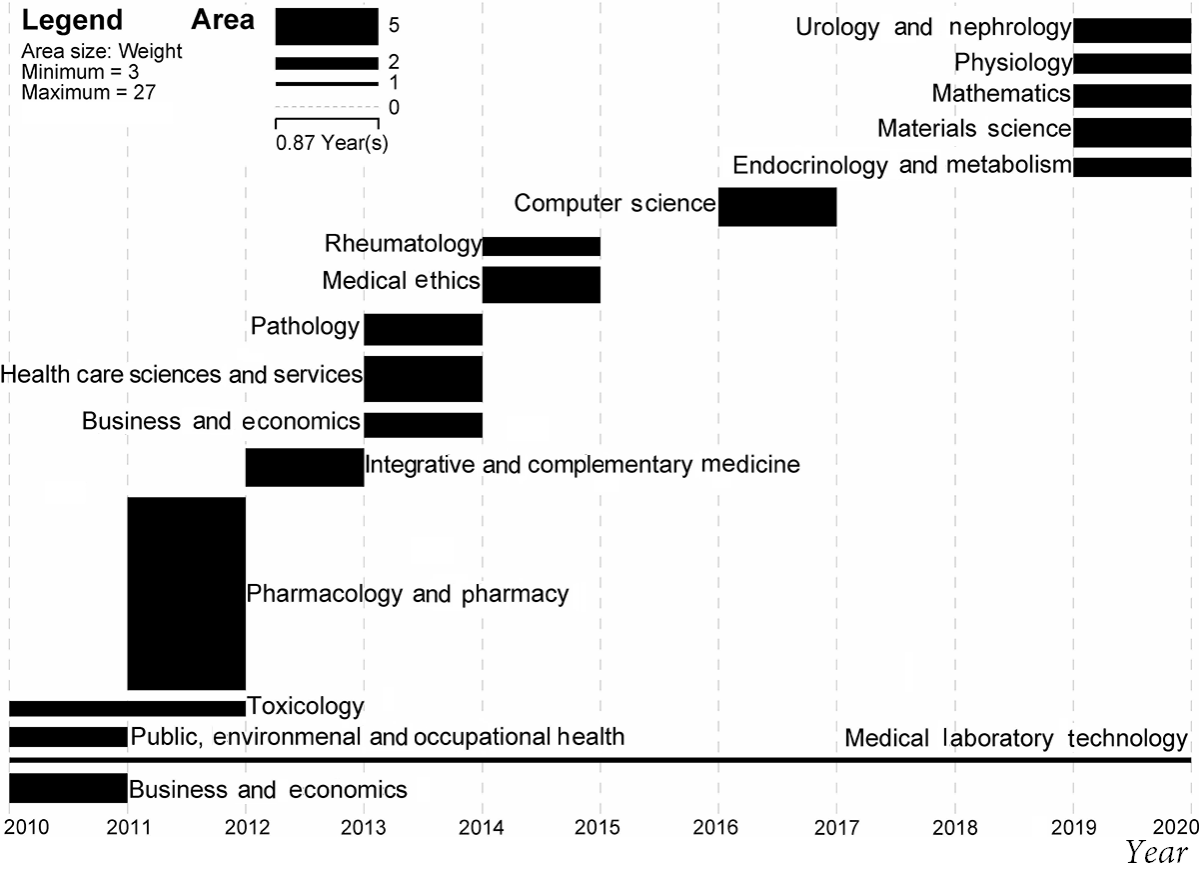
Burst disciplines of precision medicine research from 2010 to 2019.

Through the calculation of the burst intensity of the discipline, we discovered that the disciplines involved in precision medicine research have changed every year; that is, every year new disciplines enter the main positions of precision medicine research. As shown in Figure 3, the length of the horizontal bar represents the burst duration of the discipline, and its area represents the relative intensity of its burst. From the figure, we can see that the disciplines of pharmacology and pharmacy, medical laboratory technology, health care sciences and services, computer science, integrative and complementary medicine, medical ethics, pathology, toxicology, and business and economics have recently emerged in precision medicine research. In other words, precision medicine research mainly focuses on the above subjects.

### Interdisciplinary Network

#### Network Indicators of Interdisciplinary Structure

The interdisciplinary network of precision medicine research is the largest connected subgraph, which contains 433 edges, representing interdisciplinary cooperation. It is worth noting that the intensity of interdisciplinary cooperation (co-occurrence frequency) varies from 1 to 104. Cooperation, which has a co-occurrence frequency greater than or equal to 30, accounts for 51.4% of the total (2292/4458) and mainly focuses on medical informatics, cell biology, health care, sciences and services, biochemistry and molecular biology, and genetics and heredity (as shown in Table 2). This means that the interdisciplinary cooperation mentioned above is the mainstream of current precision medicine research.

**Table 2:** Disciplines with co-occurrence frequency equal to or greater than 30.

Item	Subject category	Sum of interdisciplinary frequency
1	Medical informatics	169
2	Cell biology	166
3	Health care sciences and services	162
4	Biochemistry and molecular biology	143
5	Genetics and heredity	104
6	Biotechnology and applied microbiology	104
7	Oncology	99
8	Neurosciences and neurology	98
9	Psychiatry	98
10	Mathematical and computational biology	94
11	Mathematics	94
12	Research and experimental medicine	93
13	General and internal medicine	93
14	Chemistry	76
15	Computer science	75
16	Business and economics	68

According to the overall network indicators of interdisciplinary cooperation (Table 3), the density value indicates that the discipline cooperation of current precision medicine research is poor; the cross-cutting nature of precision medicine research is still immature. At the same time, the degree centralization and closeness centralization of the network are not high, showing that the concentration of discipline cooperation in precision medicine research is not high and is scattered. In other words, the influence or dominance of a discipline on the whole cooperation network is not obvious. Network betweenness centralization is high, indicating that most interdisciplinary cooperation is likely to be indirect; that is, interdisciplinary cooperation requires other disciplines as a “bridge”. This makes the distance between two disciplines in the cooperative network long and makes the discipline cooperation network loose. The above results are also reflected in the clustering coefficient, the value of which is higher than the overall degree centralization. This means that the disciplines are gathered into different clusters due to the different cooperation structures, and interdisciplinary cooperation within the clusters is above the overall level. Therefore, we can speculate that in some subject directions, precision medicine has formed a relatively stable multidisciplinary cooperation, and researchers in various disciplines have reached a basic consensus on certain directions.

**Table 3:** Indicators of interdisciplinary networks in precision medicine research.

Indicator	Value
Number of Nodes	75
Number of Lines	433
Average Degree	11.5467
Density	0.156
Network All Degree Centralization	0.3117
Network All Closeness Centralization	0.3337
Network Betweenness Centralization	0.1249
Network Clustering Coefficient	0.3863

In the disciplinary cooperation network, the network indicators (degree centralization, closeness centralization, and betweenness centralization) of each discipline reflect its position and role in the entire network. As shown in Table 4, pharmacology and pharmacy, oncology, genetics and heredity, biochemistry and molecular biology, neurosciences and neurology, engineering, research and experimental medicine, biotechnology and applied microbiology, and cell biology have higher network indexes, indicating that these disciplines are at the core of the network. They are the most cooperative, their cooperative patterns are direct, and their cooperation is in short paths. It can be suggested that these disciplines play a leading role in current precision medicine research, and cooperation among these disciplines is the mainstream of current precision medicine research. In contrast, except for the higher betweenness centralization of pharmacology and pharmacy, the betweenness centralization of other disciplines is low. This indicates that pharmacology and pharmacy has played an important “bridging” role in the interdisciplinary cooperation of precision medicine research.

**Table 4:** Top 10 subject categories in terms of degree, betweenness, and closeness centrality in precision medicine research.

Ranking	Subject category	Degree centrality	Subject category	Closeness centrality	Subject category	Betweenness centrality
1	Pharmacology and Pharmacy	34	Pharmacology and Pharmacy	0.6379	Pharmacology and Pharmacy	0.139
2	Oncology	30	Oncology	0.6115	Oncology	0.0989
3	Biochemistry and Molecular Biology	28	Biochemistry and Molecular Biology	0.6016	Neurosciences and Neurology	0.0948
4	Genetics and Heredity	28	Genetics and Heredity	0.6016	Genetics and Heredity	0.0768
5	Engineering	26	Neurosciences and Neurology	0.6016	Engineering	0.0537
6	Neurosciences and Neurology	26	Research and Experimental Medicine	0.5781	Surgery	0.0468
7	Research and Experimental Medicine	25	Engineering	0.5736	Biochemistry and Molecular Biology	0.046
8	Biotechnology and Applied Microbiology	24	Biotechnology and Applied Microbiology	0.5649	Biotechnology and Applied Microbiology	0.0443
9	Cell Biology	23	Cell Biology	0.5606	Cell Biology	0.0433
10	Immunology	21	Immunology	0.5564	Research and Experimental Medicine	0.0408

#### Interdisciplinary Community

The disciplinary cooperation network of precision medicine research is well divided into 7 communities, with a module degree of 0.4343. This indicates the strong preference for disciplinary cooperation in precision medicine research. We discover some research directions of precision medicine with close cooperation of multiple disciplines. There is a significant difference between these directions represented by communities. As shown in Table 5, the disciplinary cooperation network for precision medicine research includes the following 5 communities:C1-oncology community, including cardiovascular system and cardiology; cell biology; respiratory system; radiology, nuclear medicine, and medical imaging; hematology; gastroenterology and hepatology, etc; C2-pharmacology and pharmacy community, including neurosciences and neurology; psychiatry; endocrinology and metabolism; toxicology; psychology; integrative and complementary medicine, etc; C3-health care sciences and services community, including mathematical and computational biology; computer science; medical informatics; engineering; public, environmental, and occupational Health, etc; C4-genetics and heredity community, including biochemistry and molecular biology; biotechnology and applied microbiology, etc; C5-biomedical social sciences; C6-research and experimental medicine; C7-immunology communities.

From the perspective of the scale of cooperation, the current disciplinary cooperation of precision medicine research is clearly divided into three levels: the largest is the C1 direction, followed by the C2, C3, and C4 directions, while the C5, C6, and C7 directions are smaller. In other words, in the past decade, precision medicine research has focused on oncology, pharmacology and pharmacy, health care sciences and services, and genetics and heredity, which represent the mainstream direction of research. However, biomedical social sciences, research and experimental medicine, and immunology research in the discipline are still weak.

**Table 5:** Interdisciplinary communities of precision medicine research.

Community; number of subject categories	Subject categories
C1; 22	Oncology; cardiovascular system and cardiology; cell biology; respiratory system; radiology, nuclear medicine and medical imaging; hematology; gastroenterology and hepatology; urology and nephrology; pediatrics; surgery; obstetrics and gynecology; rheumatology; physiology; nursing; dermatology; transplantation; otorhinolaryngology; dentistry, oral surgery and medicine; optics; reproductive biology; sport sciences; orthopedics
C2; 14	Pharmacology and pharmacy; neurosciences and neurology; psychiatry; endocrinology and metabolism; toxicology; psychology; integrative and complementary medicine; geriatrics and gerontology; nutrition and dietetics; substance abuse; education and educational research; veterinary sciences; food science and technology; anesthesiology
C3; 12	Health care sciences and services; mathematical and computational biology; computer science; medical informatics; engineering; public, environmental and occupational health; mathematics; business and economics; life sciences and biomedicine - other topics; information science and library science; telecommunications; environmental sciences and ecology
C4; 10	Genetics and heredity; biochemistry and molecular biology; biotechnology and applied microbiology; chemistry; science and technology - other topics; materials science; biophysics; physics; instruments and instrumentation; electrochemistry
C5; 7	Biomedical social sciences; social sciences - other topics; medical ethics; government and law; history and philosophy of science; social issues; legal medicine
C6; 6	Research and experimental medicine; general and internal medicine; pathology; medical laboratory technology; ophthalmology; anatomy and morphology
C7; 4	Immunology; allergy; microbiology; infectious diseases

#### Visualization of the Interdisciplinary Network

**Figure 4:**
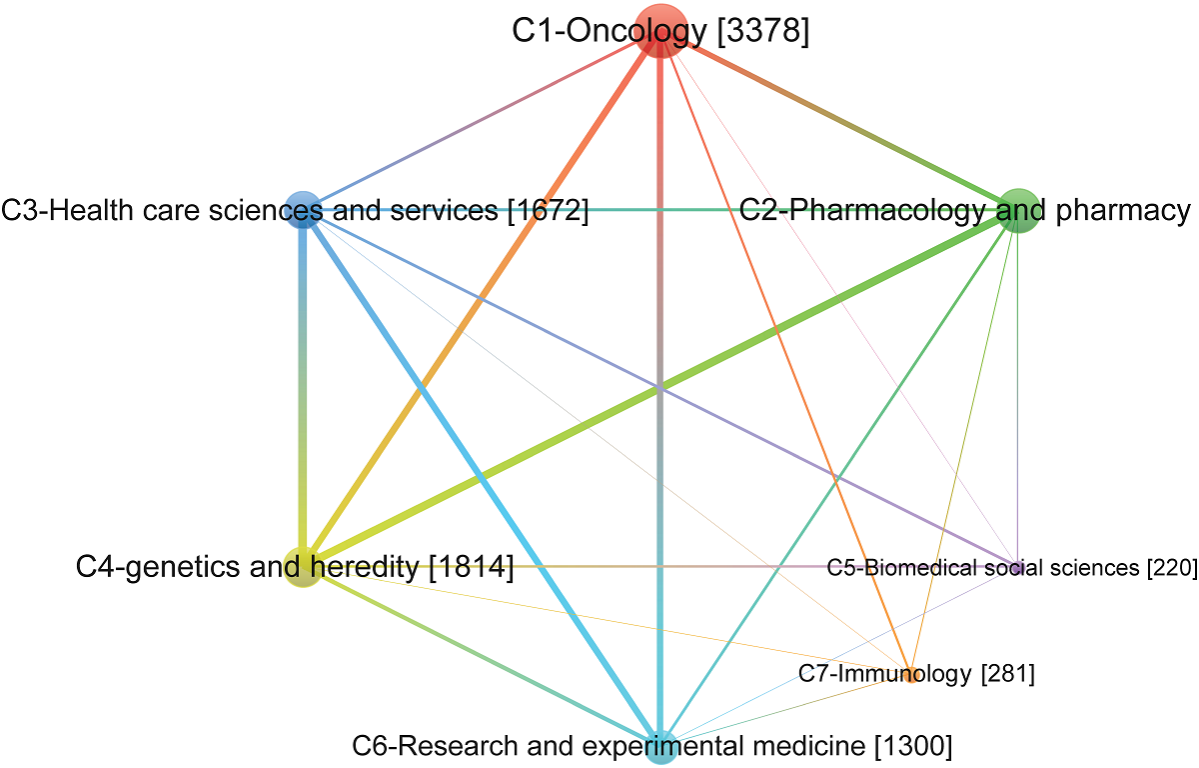
Interdisciplinary structure of communities in precision medicine research.

**Figure 5:**
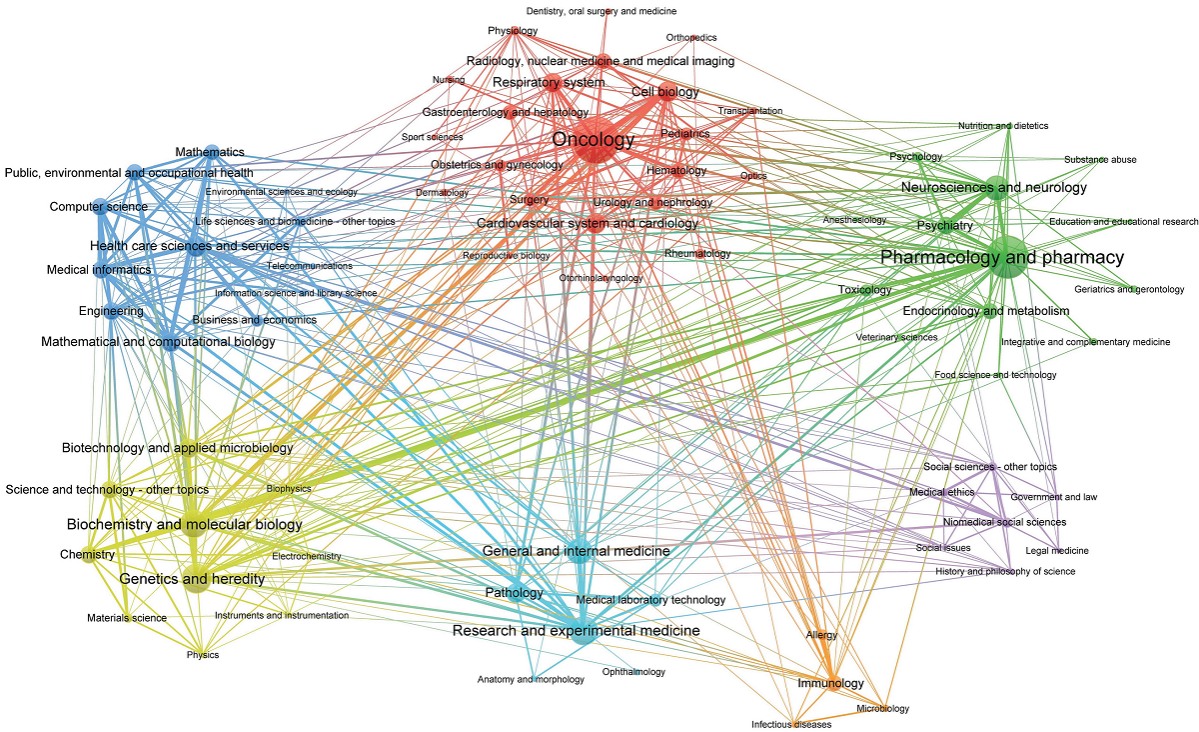
Interdisciplinary structure of all disciplines in precision medicine research.

The interdisciplinary structure of precision medicine research needs to be presented through a visual map, as shown in Figures 4 and 5. Figure 4 presents a network of disciplinary cooperative communities. Each point represents a community and is distinguished by different colors. The size of the points represents the sum of the frequencies of all disciplines in the community (the number in brackets in the figure), which can be regarded as the scale of the discipline cooperation. The larger the node is, the larger the scale of cooperation is. Each edge represents the cooperative relationship between the communities. The thickness of the edge represents the intensity of cooperation, which is proportional to the sum of the number of co-occurrences between the two communities. Moreover, there is extensive cooperation between the C1-oncology community, the C3-health care sciences and service community, and the C6-research and experimental medicine community, as well as cooperation between the C1-oncology community and the C2-pharmacology and pharmacy community. However, C5-biomedical social sciences and C7-immunology are relatively isolated and less collaborative with other communities.

Figure 5 shows the characteristics of cooperation among the internal disciplines of each community. In Figure 5, different colors indicate their own community; each node represents a discipline, and its size is proportional to the frequency of the discipline. Each edge represents the relationship between disciplines, and the thickness of the edge represents the number of co-occurrences between disciplines. This indicates that precision medicine research is inclined to concentrate on disciplinary cooperation. The network indicators of the disciplinary cooperation communities shown in Table 6 also support our conclusions presented above.

The density of each community exceeds the overall network. While the C5-biomedical social sciences, C6-research and experimental medicine, and C7-immunology communities are too small to be comparable, other communities have higher densities. In particular, the C4-genetics and heredity community has a relatively high degree of centrality and density, indicating that in precision medicine studies, the disciplines involved in this community and their related research directions are the core of precision medicine research, and they are widely related to other disciplines and research directions. Moreover, the communities of C1-oncology, C2-pharmacology and pharmacy, and C3-health care sciences and services are the main and core components of current precision medicine research disciplines and have a greater impact on the entire study.

**Table 6:** Interdisciplinary communities of precision medicine research.

Community	Number of nodes	Number of lines	Total frequency	Average degree	Density
C1-Oncology	22	53	3378	10.0455	0.2294
C2-Pharmacology and pharmacy	14	25	2245	10.6429	0.2747
C3-Health care sciences and services	12	34	1672	13.1667	0.5152
C4-Genetics and heredity	10	30	1814	16.2	0.6667
C5-Biomedical social sciences	7	16	220	9.71429	0.7619
C6-Research and experimental Medicine	6	8	1300	11.6667	0.5333
C7-Immunology	4	4	281	9.5	0.6667

### Interdisciplinarity of Precision Medicine Research

**Figure 6:**
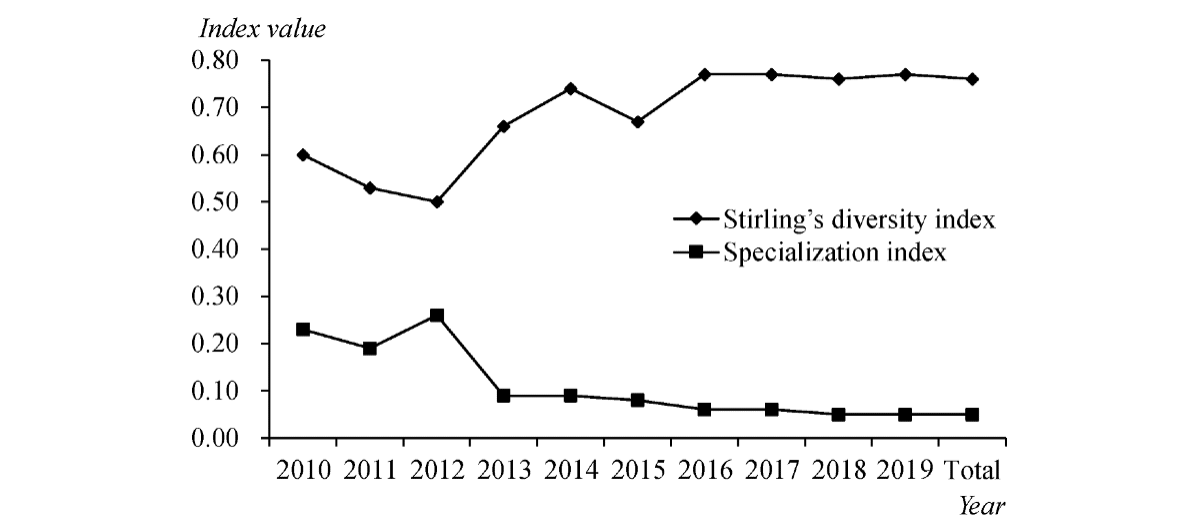
Stirling’s diversity index and the specialization index over time.

The results of Stirling's diversity index and the specialization index are shown in Figure 6. Since 2010, Stirling's diversity index values have all been above 0.5, indicating that the interdisciplinary level of precision medicine research is high. However, the specialization index has been at a low level since 2013, which shows that precision medicine research involves increasingly diversified disciplines. In general, precision medicine research has been strengthened every year by disciplinary cooperation; that is, precision medicine research is more diversified and less concentrated.

**Figure 7:**
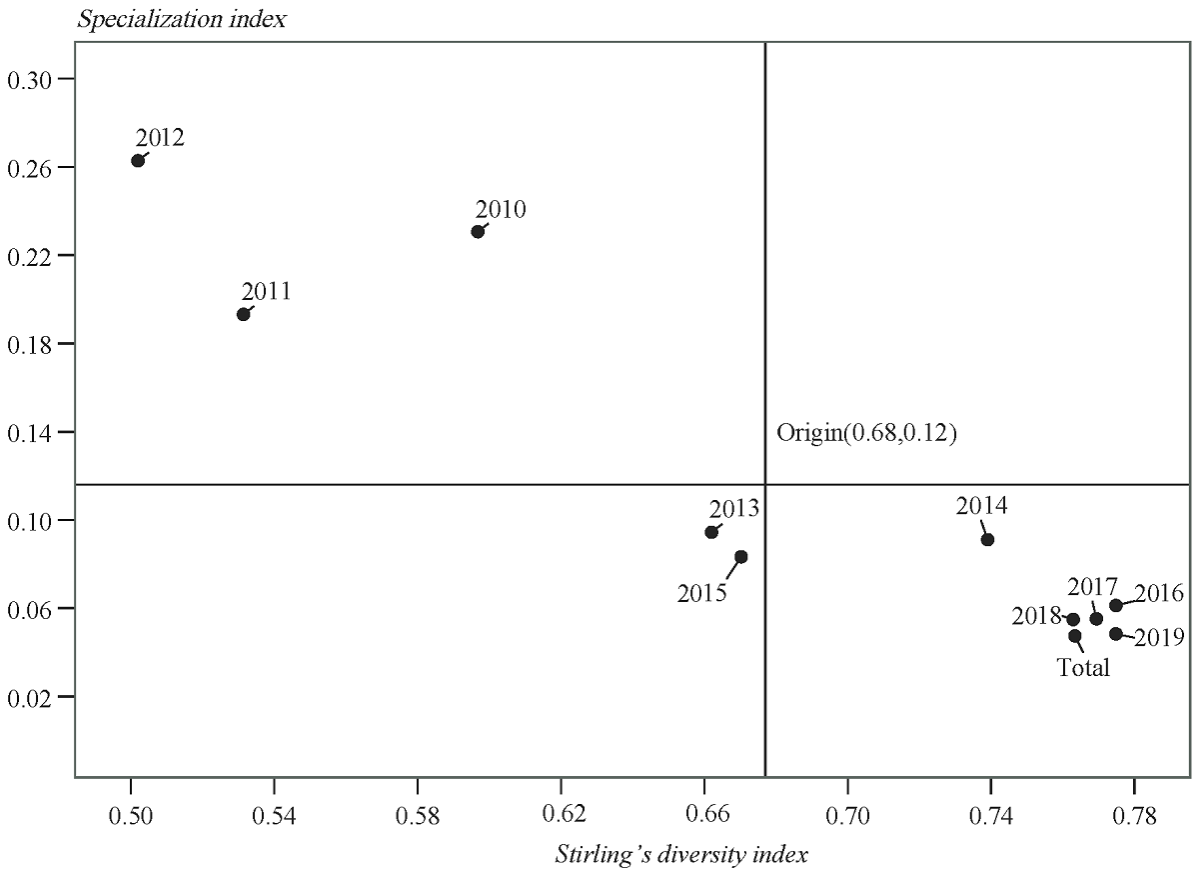
The relative status of interdisciplinarity for each year and all years combined.

Based on Stirling's diversity index and the specialization index, we have drawn a 2-dimensional map of the interdisciplinary distribution of precision medicine research for every year and we have divided each year into 4 quadrants with the average of the 2 targets as the origin to reveal the relative state of interdisciplinary cooperation in precision medicine research (Figure 7). From 2010 to 2012, precision medicine research focused on definite disciplines, but the degree of interdisciplinary cooperation was low. The figure shows that 2013 was a demarcation line for interdisciplinary cooperation in precision medicine research. With increasing concentration and diversity of precision medicine research, especially after 2014 (with the exception of 2015), the interdisciplinary cooperation of precision medicine research remained stable at a high level, which also indicates that precision medicine research is mature in interdisciplinary cooperation.

### Evolution and Trends of Interdisciplinary Collaboration

#### Background

**Figure 8:**
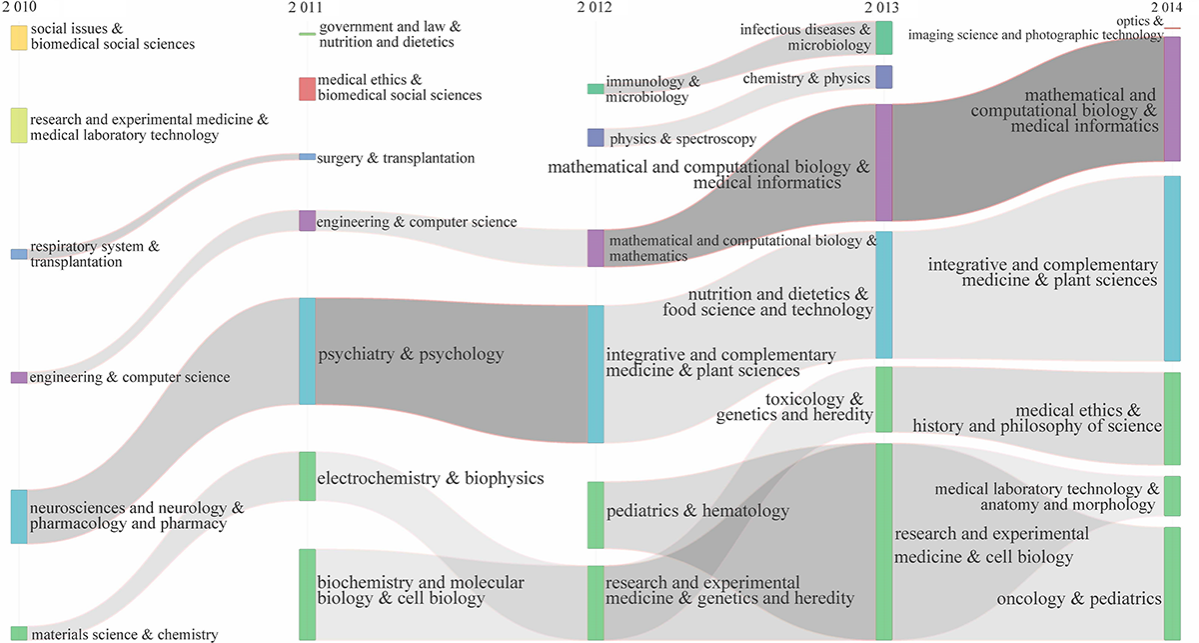
Evolution of interdisciplinary collaboration of PM research over time (2010-2014). The column represents a special interdisciplinary research, the interdisciplinary fields are distinguished by the color of columns, and the size of the column represents the scale of the special interdisciplinary research. The continuity of the column crossing the years indicates the continuity of the interdisciplinary research.

**Figure 9:**
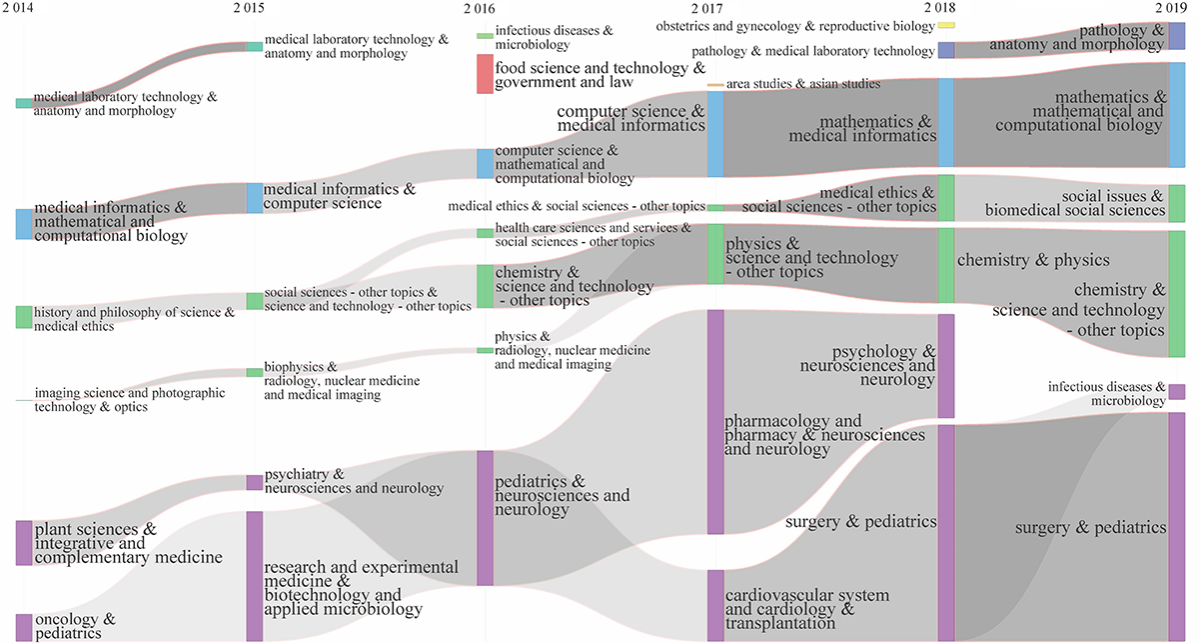
Evolution of interdisciplinary collaboration of precision medicine research over time (2014-2019). The column represents a special interdisciplinary research, the interdisciplinary fields were distinguished by the color of columns, and the size of the column represents the scale of the special interdisciplinary research. The continuity of the column crossing the years indicates the developmental continuity of the interdisciplinary research.

**Figure 10:**
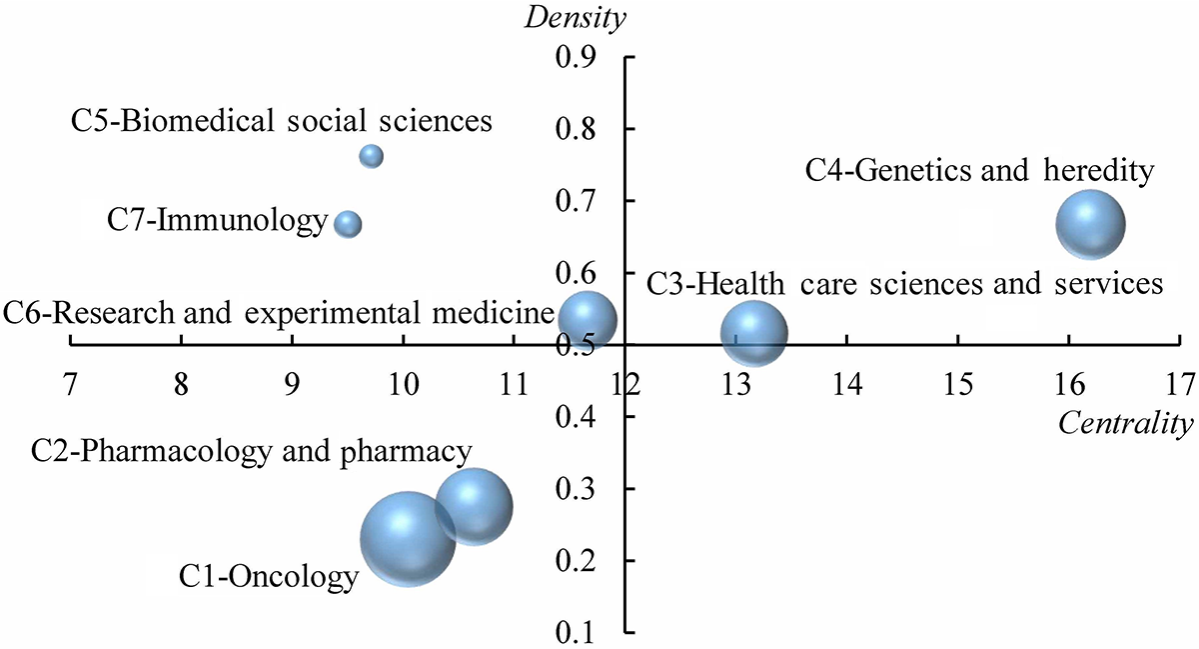
Strategic diagram of 7 interdisciplinary communities. The nodes represents the interdisciplinary communities of precision medicine, and node size is proportional to its scale. The position of the interdisciplinary communities in the graph is determined by the density and centrality, which indicate their relative position and maturity in the interdisplinary cooperation network.

Although the interdisciplinary cooperation mode changes every year, interdisciplinary cooperation continues. Considering the different scales and levels of interdisciplinary cooperation in different stages, we attempt to reveal the evolution of interdisciplinary cooperation in precision medicine research in two stages: 2010-2014 and 2015-2019 (as shown in Figure 8 and Figure 9). To show the time continuity, the second stage of the evolutionary graph starts in 2014. According to the centrality and density of the interdisciplinary cooperation community, we mapped the interdisciplinary cooperation community to the 2-dimensional strategic map with the average value of the centrality and density of all communities as the quadrant origin to reveal the relatively low position and development trend of the interdisciplinary community in precision medicine research (Figure 10).

#### Streams of Interdisciplinary Collaboration

In the first stage (2010-2014), the interdisciplinary cooperation of precision medicine research is relatively stable. There are 3 evolutionary streams: (1) mathematical and computational biology and medical information, including engineering, computer science, and mathematics; (2) integrated and comprehensive medicine and plant sciences, including neurosciences and neurology, pharmacology and pharmacy, psychiatry, psychology, nutrition, dietetics, food science and technology; and (3) research and experimental medicine and cell biology, including materials science, chemistry, biochemistry and molecular biology, cell biology, pediatrics, physiology, genetics and condition, toxicology, medical history, history and philosophy of science, and oncology.

The continuity of each stream of cooperation is good, but the scales are different. It can also be observed that the interdisciplinary cooperation of precision medicine research at this stage focuses on the two main streams: (1) integrative and complementary medicine and plant sciences and (2) research and experimental medicine and cell biology. It is worth noting that research and experimental medicine and cell biology have been fused and differentiated many times. For example, in 2013, pediatrics and hematology, research and experimental medicine, and genetics and heredity were fused into another stream, research and experimental medicine and cell biology. Interestingly, in 2014, the streams of research and experimental medicine and cell biology were also fused into the completely new streams of medical laboratory technology, anatomy and morphology and oncology and pediatrics. The characteristics of the trends in disciplinary evolution show the instability and expansion of interdisciplinary cooperation in precision medicine research.

In the second phase (2015-2019), according to the interdisciplinary cooperative community division in 2014 (Figures 8 and 9), the interdisciplinary cooperation at this stage continued the status of the first phase and continued to evolve into 2019. At this stage, there are three major evolutionary streams. First, medical informatics and mathematical and computational biology already has a relatively stable relationship with computer science and other disciplines. Second, history and philosophy of science and medical ethics, including social sciences, science and technology, chemistry, physics, health care sciences and services, medical ethics, social issues, and biomedical social sciences, is integrated with imaging science and photographic technology, optics, and physics. Finally, plant sciences and integrative and complementary medicine and the context of oncology and pediatrics is a continuation and fusion of the previous phase, including research and experimental medicine, biotechnology and applied microbiology, neurosciences and neurology, pharmacology and pharmacy, cardiovascular system and cardiology, transplantation, psychology, and surgery.

At this stage, interdisciplinary cooperation focused on the fused stream of plant sciences and integrative and complementary medicine and oncology and pediatrics. It is worth noting that pharmacology and pharmacy and neurosciences and neurology (2017) and surgery and pediatrics (2018) became the two major communities in the stream mentioned above. This indicates a shift in the direction of interdisciplinary collaboration in precision medicine research.

At the same time, there are a few isolated communities or intermittent evolutionary streams, such as immunology and microbiology, physics and spectroscopy, food science and technology, and government and law. These streams or communities do not effectively continue. These changes are worth considering in precision medicine interdisciplinary collaboration.

#### Development Trends of Interdisciplinary Collaboration

According to the indicators of community centrality and density, the developmental trend of the interdisciplinary cooperative community of precision medicine research is shown in Figure 10. C3 and C4 are in the first quadrant. Due to their high centrality and density, we can speculate that these communities are the core of interdisciplinary cooperation in precision medicine research, and their cooperative status is relatively stable and mature. C5, C6, and C7 are in the second quadrant. Although their cooperative state is relatively stable and mature, the disciplines involved are no longer the core of current precision medicine research. It is worth noting that the location of the C6 community is very close to the original point and has great potential for development. It is likely to be the core community in the future. C1 and C2 are in the third quadrant as the two largest cooperative communities. Their cooperative state is unstable and mature, and it is not the core discipline or direction of the entire precision medicine research.

## Discussion

### Principal Findings

In this study, we confirm the unbalanced state of disciplinary distribution and clarify the immature collaboration network. In the community research, the communities representing the major cooperation directions in the precision medicine field were elaborated. Community 4 is the most central and cooperative in the interdisciplinary network. Ultimately, we successfully predicted the future directions of cooperation in precision medicine in the collaboration strategy map.

First, we found that the disciplines involved in precision medicine are comprehensive; up to 105 disciplines are included, and this number is increasing yearly, indicating that subject collaboration in precision medicine is still developing. However, the frequency of disciplines involved in collaboration in precision medicine is heterogeneous. Considering the frequency of disciplinary collaborations, cross-disciplinary collaboration in precision medicine is mainly focused on clinical disciplines such as oncology, neurology, and cardiology and technology-associated disciplines such as pharmacology, genetics, and molecular biology. These disciplines are the main pillars in the current precision medicine field, indicating that the current stage of precision medicine is based first on molecular biology and genetics technology with pharmacology and pharmacological genomics and is later applied to clinical disciplines. In addition, we discovered that some emerging disciplines continuously joined the collaborative network of precision medicine, such as environment and occupation, business and economics, medical ethics, medical informatics, and computer science. The emergence of these disciplines indicates that the knowledge system of precision medicine is constantly being enriched, the depth and breadth of scientists' understanding is constantly improving, and the research topics and methods are being diversified. These factors have promoted the development of precision medicine.

Second, we conducted further analysis of the overall disciplinary collaboration network of precision medicine. Through co-occurrence frequency analysis, we discovered the uneven status of disciplinary collaboration. It can be speculated that medical informatics is the protagonist of the disciplinary collaboration network of precision medicine. According to the overall network index of disciplinary cooperation, we found the following characteristics of the network: (1) disciplinary cooperation in precision medicine is not yet mature; (2) disciplinary cooperation is decentralized; (3) the leading disciplines are absent in the overall cooperation network; (4) and the pattern of disciplinary cooperation is mostly indirect rather than direct. It is worth noting that some important disciplines in the network, such as pharmacology and pharmacy and oncology, play a “bridging” role in disciplinary cooperation.

Third, the communities in the disciplinary cooperation network are the cluster of disciplines with close cooperation, representing a certain research direction. Through the community division, we find that the field of precision medicine has formed several well-developed research directions. The size of these communities varies, partly reflecting the different maturity among research directions in precision medicine. In the study of community visualization, we propose the following two laws: (1) like disciplines, collaboration between communities is equally unbalanced. It is worth noting that the C4 community is most active in the disciplinary cooperation network, which suggests that the C4 community is in the core position of the entire disciplinary cooperative network. This might be due to the importance of disciplines within the C4 community, such as genetics and heredity. These disciplines provide fundamental technologies that are widely adopted or used in precision medicine. It is thus assumed that the C4 community symbolizes a relatively mature direction in precision medicine. (2) In the disciplinary cooperation network, disciplinary cooperation is significantly higher within the community than among the communities. This may be due to the initial stage of some interdisciplinary research directions; whose application is not yet mature enough to affect other communities. Furthermore, we cannot exclude the influence of the researcher's limited vision such that some valuable interdisciplinary issues have not received much attention. In addition, there are obvious time nodes in the history of disciplinary cooperation in precision medicine. The trend toward multidisciplinary collaboration increased after 2010 and levelled off in 2014.

Fourth, we presented the history of the disciplinary cooperation of precision medicine in evolutionary research. According to the evolutionary map, the disciplinary cooperation of precision medicine can be divided into two stages with 2014 as the time node: (1) the initial stage is 2010-2014, which involves three well-developed evolutionary contexts: medical informatics, integrated medicine, and molecular biology. We can speculate that integrated medicine, molecular genetics, mathematics and computer science (big data processing) are the three major research directions at this stage. They built the fundamental knowledge system of precision medicine. In Phase II (2015-2019), we can identify four complete evolutionary contexts: medical informatics and computer science, social sciences, imaging and physical chemistry, and clinical medicine. According to the evolutionary contexts, we can identify the following trends in the cooperative development of precision medicine disciplines. (1) Medical informatics and computer science is regarded as an important and continuous research direction due to the continuing and urgent requirements of big data processing. (2) In addition to traditional molecular biology, molecular imaging based on physics, chemistry, and radiology has become a new method for exploring biomarkers. (3) The participation of social sciences, such as philosophy, law, and ethics, has enriched the humanities in precision medicine. (4) An increasing number of clinical disciplines, such as oncology, pediatrics, cardiovascular medicine, and psychiatry, were merged into the disciplinary collaboration network. This indicates that precision medicine is so mature that its application spread from oncology to other clinical subjects.

In our strategic diagram study, we assessed the maturity and trends of communities in the discipline cooperative network. We found that C3-medical informatics and computer science and C4-genetics and molecular biology are the core of the discipline community network, which indicates that the big data processing of biology is a stable, core research direction. It is worth noting that in noncore disciplines, pathology and anatomy in the C6 community have the core potential to become an interdisciplinary network. With the progress of the C6 community, the precision medicine field will be pushed forward. C1-oncology and C2-pharmacology will remain in a noncore position. This does not mean that oncology and pharmacology are not important but points instead to the increasing application of precision medicine in clinical medicine and the wider range of research in other directions. This can be considered a major sign of the maturity of precision medicine.

In conclusion, the findings of this study can help researchers understand the entire network of precision medicine disciplines, clarify the main research direction, and predict future trends. This work is of reference value to scientists and clinical experts to determine future work in precision medicine research.

### Limitations and Future Study

The study has some obvious limitations. First, we adopted the Web of Science Core Collection database as the only data source, which may cause some bias. However, the data collected from the SCI database could represent the trends and evolution of the precision medicine field. On the one hand, the Web of Science Core Collection is a typical database that contains subdatabases such as SCI-EXPANDED, SSCI, and A&HCI. The SCI database includes 8600 core journals, SSCI contains 3100 core journals, and A&HCI contains 1700 core journals. Furthermore, most high-level publications in precision medicine are available in the Web of Science Core Collection database. However, some important and well-known databases, such as PubMed, Embase, and CSA, were not chosen, leading to inevitable biases. Second, the development of precision medicine research is dynamic as a result of time limits. After a definite period, the nature of the interdisciplinary collaboration will change. The conclusions may not be accurate after a certain amount of time. Taking these limitations into account, we will continue to update the data of this research regularly. Finally, the variations in development in different regions remain unknown. We will also further examine the regional variation in precision medicine development.
